# Galectin-3 Plays an Important Role in BMP7-Induced Cementoblastic Differentiation of Human Periodontal Ligament Cells by Interacting with Extracellular Components

**DOI:** 10.1155/2023/5924286

**Published:** 2023-06-23

**Authors:** Min-Jeong Choi, Tae Min You, Young-Joo Jang

**Affiliations:** ^1^Department of Nanobiomedical Science and BK21 FOUR NBM Global Research Center for Regenerative Medicine, Dankook University, Cheonan 31116, Republic of Korea; ^2^Department of Advanced General Dentistry, School of Dentistry, Dankook University, Cheonan 31116, Republic of Korea; ^3^Department of Oral Biochemistry, School of Dentistry, Dankook University, Cheonan 31116, Republic of Korea

## Abstract

Human periodontal ligament stem cells (hPDLSCs) contain multipotent postnatal stem cells that differentiate into PDL progenitors, osteoblasts, and cementoblasts. Previously, we obtained cementoblast-like cells from hPDLSCs using bone morphogenetic protein 7 (BMP7) treatment. Differentiation into appropriate progenitor cells requires interactions and changes between stem or progenitor cells and their so-called environment niches, and cell surface markers play an important role. However, cementoblast-specific cell surface markers have not yet been fully studied. Through decoy immunization with intact cementoblasts, we developed a series of monoclonal antibodies against cementoblast-specific membrane/extracellular matrix (ECM) molecules. One of these antibodies, the anti-CM3 antibody, recognized an approximate 30 kDa protein in a mouse cementoblast cell line, and the CM3 antigenic molecule accumulated in the cementum region of human tooth roots. Using mass spectrometric analysis, we found that the antigenic molecules recognized by the anti-CM3 antibody were galectin-3. As cementoblastic differentiation progressed, the expression of galectin-3 increased, and it localized at the cell surface. Inhibition of galectin-3 via siRNA and a specific inhibitor showed the complete blockage of cementoblastic differentiation and mineralization. In contrast, ectopic expression of galectin-3 induced cementoblastic differentiation. Galectin-3 interacted with laminin *α*2 and BMP7, and these interactions were diminished by galectin-3 inhibitors. These results suggested that galectin-3 participates in binding to the ECM component and trapping BMP7 to induce, in a sustained fashion, the upregulation of cementoblastic differentiation. Finally, galectin-3 could be a potential cementoblast-specific cell surface marker, with functional importance in cell-to-ECM interactions.

## 1. Introduction

The dental cementum is a mineralized layer covering the dentin surface and anchors fibrous connective ligament tissues on tooth-root surfaces [1]. Cells with the ability to form cementum are called cementoblasts, which are formed from highly differentiated mesenchymal cells found in periodontal ligament tissue. Cementoblasts are differentiated from multipotent postnatal stem cells located in the human periodontal ligament [2, 3]. Given that periodontal ligament tissue maintains the unmineralized fibrous state under physiological conditions, osteogenic/cementogenic differentiation is generally prevented, and ligament fibroblast differentiation usually occurs predominantly in periodontal ligament stem cells [4–6]. How cementoblast differentiation for the periodontal connective hard tissue is determined has not yet been fully resolved, but differentiation factors and ligands are basically the first determinants of specific differentiation of stem cells. Among the BMP family members, BMP7 was shown to stimulate osteoblastic differentiation in murine bone marrow stromal cells, human adipose-derived stem cells, and human dermal-derived fibroblast cells through the Smad and/or MAPK pathways [7–9]. Based on previous histologic data using in vivo models of periodontal development, BMP7 is expressed during cementogenesis [10, 11]. Additionally, BMP7 induces the expression of cementogenic markers in both normal and immortal periodontal ligament stem cells of murine and human through a mechanism different from that for osteogenic and odontogenic differentiation [12–14]. Recombinant BMP7 reduces TGF-*β*1-mediated profibrotic effects during fibrogenesis [15], and cementoblastic gene expression was completely decreased by TGF-*β*1, whereas it was significantly increased when cells were treated with BMP7 and the TGF-*β* type I receptor inhibitor [4]. BMP7 regulates mineralized tissue-associated genes in cementoblasts *in vitro* and influences the expression profile of cementoblast extracellular matrix components and cell adhesion molecules [14, 16].

Galectin-3 is a protein belonging to the family of the *β*-galactoside-specific lectins, and it has been recognized as a modulator of several biological functions inside and outside the cell [17, 18]. Galectin-3 has been known to be involved in cell-cell and cell-matrix adhesion, cell proliferation and differentiation, and metastasis [19–21]. During osteogenic differentiation, galectin-3 is upregulated as the osteoblast markers and is essential for bone cell maturation and function [22, 23]. The osteogenic differentiation of human bone marrow mesenchymal stem cells is suppressed by galectin-3 knockdown [24]. The expression of Runt-related transcription factor 2 (Runx2) shows a positive correlation with galectin-3 during osteoblastic development, and an increase of galectin-3 induces levels of Runx2 and alkaline phosphatase (ALP) in mesenchymal stem cells, suggesting that galectin-3 is not only a downstream target but an upstream factor of the main osteogenic regulator Runx2 [25, 26]. Though galectin-3 is a positive regulator for osteoblast differentiation, exogenous galectin-3 inhibits osteogenesis of a human preosteoblast cell line, suggesting that galectin-3 might influence bone homeostasis by regulating the function and/or interplay of osteoblasts and osteoclasts [27, 28].

Recently, we developed a set of monoclonal antibodies against the membrane and ECM molecules of cementoblast-like cells. In this study, we present a novel IgG-type antibody, the anti-CM3 antibody, to identify the cementoblast-specific antigen, galectin-3. Coincidentally, in our recently published paper, we identified galectin-3 as one of the genes specifically expressed in BMP7-induced human cementoblast-like cells through RNA-sequencing (RNA-seq) analysis and observed protein accumulation in dental cementum [16]. Since the role of galectin-3 in the differentiation of hPDLSCs into cementoblasts is not yet well known, this study was aimed at investigating a novel role of galectin-3 based on our recent findings.

## 2. Materials and Methods

### 2.1. Cell Culture

hPDLCs were obtained from adult periodontal ligament tissues according to the previously reported methods [4]. Briefly, third molars were obtained from young adult patients without systemic disease (19~23 years of age). Based on the approval of the Dankook University IRB (DKU NON 2020-008), we received approval for patients visiting Dankook Dental Hospital. The inclusion criteria were teeth with complete eruption into the oral cavity and no gingival inflammation (periodontal probing depth < 3 mm, no plaque, no bleeding on probing, and no signs of clinical loss of attachment). Teeth with gingival inflammation or mobility were excluded. An extraction forceps was placed in the cervical area of the teeth to avoid damaging the periodontal ligament, and simple extraction was performed using rotational motion and gentle force.

Periodontal ligament tissues were digested by 3 mg/mL collagenase type-1 (Millipore) and 4 mg/mL dispase (Sigma). Cells were cultured with *α*-MEM (Welgene) containing 20% fetal bovine serum (FBS, Hyclone) and antibiotics (Cytiva) at 37°C in 5% CO_2_. The independently isolated hPDLCs from 3 patients were not combined and used separately in experiments. For cementoblast differentiation, hPDLCs were cultured for a total of 9 days with treatment with 100 ng/mL BMP7 (Prospec) and 10 *μ*M SB431542 (Tocris) every 2 days. For inhibition of galectin-3 activity, hPDLCs were treated with GB1107 (3,4-dichlorophenyl 3-deoxy-3-[4(3,4,5-trifluorophenyl)-1H-1,2,3-triazol-1-yl]-1-thio-*α*-D-galactopyranoside) in the indicated concentrations. For mineralization, cells were treated with 100 nM dexamethasone (Sigma), 5 mM *β*-glycerophosphate (Sigma), and 100 *μ*M ascorbic acid (Sigma) for 14 days. OCCM-30, NIH3T3, and HeLa cells were cultured with DMEM (Welgene) containing 10% FBS and antibiotics at 37°C in 5% CO_2_.

### 2.2. Quantitative Real-Time PCR (qRT-PCR)

Total RNA extraction was performed using the AccuPrep® Universal RNA Extraction Kit (Bioneer) according to the procedure provided by the company. cDNA was synthesized from total RNA by using the ReverTra Ace™ qPCR RT kit (Toyobo). The qRT-PCR was performed using the iTaq™ Universal SYBR™ Green Supermix (Bio-Rad) in a StepOnePlus™ Real-Time PCR machine (Thermo Fisher Scientific). The primers used for qRT-PCR are shown in Table 1. The cycling parameters of qPCR were followed: 1 cycle for 30 sec at 95°C, 40 cycles for 15 sec at 95°C, and 1 min at 55~60°C. A dissociation curve was increased by 0.5°C from the range of 65°C to 95°C. The variability of an internal control in the target gene expression was normalized using the GAPDH. The threshold cycles (CT) were obtained, and the relative quantification was calculated using the 2-*ΔΔ*CT method. With regard to the *ΔΔ*CT value, the first *Δ*CT referred to the difference between the target gene and GAPDH, and the second *Δ*CT came from comparing the calculated *Δ*CT values between differentiated and undifferentiated control conditions.

### 2.3. Statistical Analysis

The paired-sample *t*-test is a statistical procedure used to compare the difference between two populations using the GraphPad Prism 6 program. For all graphs, data are represented as mean ± SD and considered statistically significant for a *P* value less than 0.05. All experiments were repeated three times (*n* = 3).

### 2.4. Construction of mAbs against the Intact Human Cementoblast-Like Cells

Production of mAbs against antigens of the intact cells was performed as previously reported with modification [29–31]. An animal study protocol was approved by the Institutional Animal Care and Use Committee of Dankook University. Briefly, cells were dissociated by using an enzyme-free dissociation solution (Millipore) and were injected into the hind footpads of 11 female BALB/c mice for immunization. BMP7-induced cementoblastic cells and hPDLCs were injected into the left and right hind footpads, respectively. After immunization, a lymphocyte suspension from the left popliteal lymph nodes was fused to FO myeloma cells (ATCC). Hybridomas were cultured in DMEM supplemented with 20% FBS (Hyclone) and HAT component (Sigma-Aldrich), and the clonal selection was performed using an enzyme-linked immunosorbent assay (ELISA) and flow cytometric analysis on the cementoblastic cells and hPDLCs.

### 2.5. Antibody Characterization

The immunoglobulin isotype of each mAb was determined using the Mouse Immunoglobulin Isotyping Kit (BD Pharmingen), according to the supplier's protocol. Rat anti-mouse IgGs, IgM, IgA, Ig*κ*, and Ig*λ* were used for coating a multiwell plate, and a hybridoma supernatant was applied into each well. The reference immunoglobulin mixtures (BD Biosciences) were used as positive controls. For antibody gene sequencing, total RNA was extracted from hybridoma cells using the easy-spin™ Total RNA Extraction kit (Intron), and cDNA was synthesized using the Maxime RT-PCR PreMix kit (Intron). To amplify the variable regions of heavy and light chains, PCR primers were used as described previously [32]. For heavy chain sequencing, two variable heavy chain forward primers were combined with an isotype-specific constant region reverse primer. For light chain sequencing, three *κ* variable light chain forward primers were combined with the corresponding constant region reverse primer.

### 2.6. Antibody Purification

The antibody was purified via Protein G Agarose column chromatography from the hybridoma culture media. Beads were incubated in the culture media and were loaded onto the column and washed with PBS. For elution of the antibody, glycine buffer (pH 2.5) was added. The eluted antibody was immediately placed in 1 M Tris-HCl (pH 9.0) for neutralization. After dialysis, it was quantified and analyzed using SDS-PAGE.

### 2.7. Flow Cytometry

Cells dissociated via the enzyme-free dissociation solution (Millipore) were incubated with proper antibodies or hybridoma supernatants in PBS containing 1% BSA on ice, followed by treatment with FITC-conjugated anti-mouse IgG (1 : 100, Santa Cruz) as the secondary antibody. Cells were analyzed using flow cytometry in FACSCalibur™ (BD Biosciences). Antibody-binding affinity was analyzed by using the CellQuest and WinMDI programs.

### 2.8. Gene Interference and Ectopic Expression

For depletion, the siRNA oligonucleotides for target genes were synthesized by the Bioneer Corporation. Cells were transfected with 100 pmol siRNA duplex using Lipofectamine RNAiMAX (Thermo Fisher Scientific) according to the manual provided by the manufacturer. After treatment with BMP7 for the first 2 days, siRNA transfection was performed every 2 days for a total of 9 days, and BMP7 was added at the same time during transfection. For overexpression of the target protein, full-length cDNAs were subcloned in pcDNA3.1(+). The ORF clone was tagged by DYKDDDDK in the C-terminus. The flanking sequences of the cloning site and full ORF sequences were confirmed via sequence analysis. Cells were transfected with plasmid DNAs using Lipofectamine® 2000 (Thermo Fisher Scientific) according to the manual provided by the manufacturer. cDNA transfection was carried out repeatedly every 2 days for 5 days.

### 2.9. Preparation of Total Cell Extract and Subcellular Fractionations

For preparation of the total cell extract, cells were lysed on ice in a lysis buffer (20 mM Tris-HCl pH 8.0, 150 mM NaCl, 1% NP40, 2 mM EDTA pH 8.0, 2 mM EGTA pH 8.0, 1 mM Na_3_VO_4_, 10 mM NaF, and 20 mM *p*-nitrophenol phosphate) containing protease inhibitors. For subcellular fraction preparation, cells were lysed by lysis buffer A (50 mM HEPES, pH 7.4, 150 mM NaCl, 25 *μ*g/mL digitonin, and 1 M hexylene glycol). After centrifugation, the supernatant was used as a cytosolic fraction. The pellet was resuspended in lysis buffer B (50 mM HEPES, pH 7.4, 150 mM NaCl, 1% NP40, and 1 M hexylene glycol). After centrifugation, the supernatant was used as a membrane fraction.

### 2.10. Western Blot Analysis and Immunoprecipitation

Cell lysates were separated on SDS-PAGE. Proteins on the gel were transferred to a PVDF membrane (Millipore) and blocked with 5% skim milk. After blocking, the membrane was incubated with a primary antibody, followed by incubation with a horseradish peroxidase- (HRP-) linked secondary antibody (Bio-Rad). To obtain optimal results, the primary antibodies were used at 200-500 ng/mL and incubated at room temperature for 3 hours or at 4°C for 16 hours. In case of the biotinylated proteins, HRP-linked streptavidin (GE HealthCare) was used. The signals were visualized by using the ECL Western Blotting Detection Kit (GE HealthCare). For immunoprecipitation, cell extracts were incubated with Protein A/G PLUS-Agarose (Santa Cruz) for preclearing. After removing the bead, cell extracts were incubated with the primary antibody, followed by pull-down with the Protein A/G PLUS-Agarose.

### 2.11. Immunocytochemistry

A coverslip was coated with 1 mg/mL fibronectin (Sigma), and cells were seeded on it. The number of cells seeded in one well containing a coverslip of the 6-well plate was 2000~10,000 cells. Cells were fixed with 4% paraformaldehyde (Sigma) at 4°C and permeabilized with Triton X-100 (0.1% Triton X-100 in PBS pH 7.4). For blocking, cells were incubated with a 10% horse serum, followed by incubation with the primary antibody. As a secondary antibody, Cy3-conjugated anti-mouse IgG or FITC-conjugated anti-rabbit IgG (Jackson ImmunoResearch) was used. FITC-/Cy3-conjugated phalloidins (Abcam) and 4,6-diamidino-2-phenylindole (DAPI) were cotreated for detection of cytoskeleton/cell boundary and nuclei, respectively. Samples were detected using the LSM700 (Carl Zeiss).

### 2.12. Immunohistochemistry

The tooth root part of the human third molar was fixed in 4% paraformaldehyde (Sigma), followed by washing in tap water. After decalcifying in a RapidCal solution (BBC Biochemical), the tissue was dehydrated with a series of graded ethanol, followed by clearing in xylene. Then, the tissue was embedded in paraffin wax. The paraffin block was sectioned with 5 *μ*m thickness using a microtome machine (RM2255, Leica). Sections were deparaffinized with xylene and hydrated with a series of graded ethanol. For antigen retrieval, sections were incubated with 0.05% trypsin and then incubated with 3% H_2_O_2_ in methanol for 20 min at RT to remove the endogenous peroxidase activity. After blocking in PBS containing 5% horse serum and 0.1% Tween 20, they were incubated with the primary antibody, followed by incubating with biotinylated anti-mouse IgG (Vector Laboratories). For detection of signals, sections were treated with a VECTASTAIN ABC Reagent (Vector Labs) and incubated with the diaminobenzidine (DAB) substrate (Vector Labs) until the desired signal was developed according to the manufacturer's instructions. Slides were mounted in the Eukitt quick-harder mounting medium (Sigma), and microscopic observation was performed using an upright microscope (Eclipse 80i, Nikon).

### 2.13. Cell Proliferation and Cytotoxicity Assay

To assess the cells' viability, cell numbers were determined via a Cell Counting Kit-8 (Dojindo Laboratories) according to the manual provided by the manufacturer. Briefly, cells were dispensed in 100 *μ*L of cell suspension (10,000 cells/well) in a 96-well plate and were incubated for an appropriate length of time (e.g., 0, 24, 48, or 72 hours). 10 *μ*L of the CCK-8 solution was added to each well of the plate. After incubation for 1 hour, the absorbance at 450 nm using a microplate reader was measured.

## 3. Results

### 3.1. BMP7 Treatment Promotes Cementoblastic Differentiation of Human Periodontal Ligament Cells

**Figure 1:**
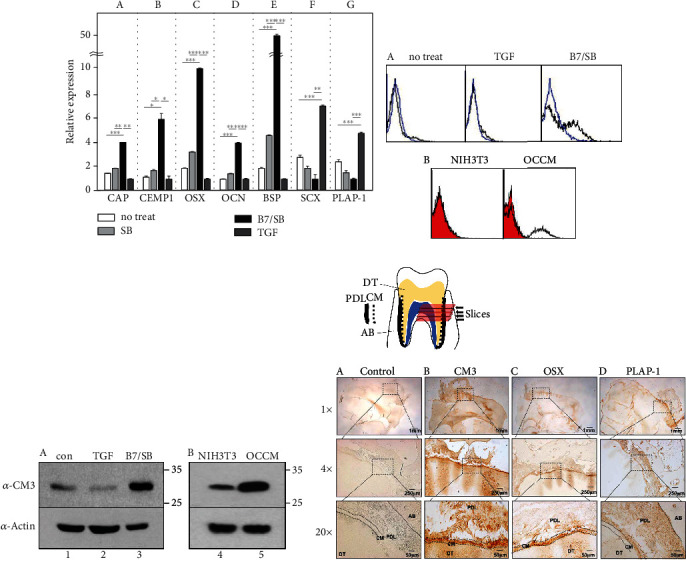
Anti-CM3 antibody binds to BMP7-induced cementoblast-like cells and cementum in tooth root. (a) Cytodifferentiation of hPDLCs into cementoblast-like cells via BMP7 treatment together with SB431542, an inhibitor of TGF-*β*1 signaling. Cementoblast-like cells were identified via increased transcriptional expression of cementoblast-specific markers such as CAP (A), CEMP1 (B), OSX (C), OCN (D), and BSP (E). Reversely, PDL fibroblastic markers such as SCX (F) and PLAP-1 (G) were upregulated in hPDLCs treated with TGF-*β*1. Statistical significance is determined using Student's *t-*test (*n* = 3). ^∗^*P* < 0.05; ^∗∗^*P* < 0.01; ^∗∗∗^*P* < 0.001. (b) Cell-binding affinity of anti-CM3 antibody using FACS analysis. Cells were detached from the culture dish under the nonenzymatic condition, and anti-CM3 antibody was added to the intact cells, followed by FITC-labeled secondary antibody. Human periodontal ligament stem cells (A) and mouse cell lines (B) were used for the antibody-binding experiment. (c) Western blot analysis of endogenous CM3 antigen in hPDLCs (A) and mouse cell lines (B). In (b, c), TGF-*β*1 and B7/SB indicate hPDLCs treated with 10 ng/mL TGF-*β*1 and hPDLCs treated with 100 ng/mL BMP7 and 10 *μ*M SB431542, respectively. OCCM and NIH3T3 indicate mouse cementoblast cells and mouse fibroblast cells, respectively. (d) Immunohistochemistry via anti-CM3 antibody. Antibodies were accumulated in the cementum of the tooth. (A) No staining control without antibody; (B) with anti-CM3 antibody; (C) with anti-OSX antibody as a positive control for the cementum; (D) with anti-PLAP-1 antibody as a negative control. AB: alveolar bone; PDL: periodontal ligament; CM: cementum; DT: dentin.

Primary periodontal ligament cells (PDLCs) cultured from human periodontal ligament tissues were used to establish cementoblast-like cells. In our previous report, we documented the culture conditions for cementoblastic differentiation from human periodontal ligament cells (hPDLCs) [4]. BMP7, a potent bone-inducing factor, was used as the main inducing factor for cementoblastic differentiation [14, 33]. A TGF-*β* type 1 receptor inhibitor, SB431542, was also used because the differentiation process of fibroblasts must be completely inhibited to achieve maximum efficiency for cementoblast differentiation [4]. The transcriptional expression of known cementoblastic markers was investigated via quantitative real-time polymerase chain reaction (qRT-PCR) analysis to confirm that hPDLCs were differentiated into cementoblast-like cells. Treatment of hPDLCs with BMP7 and SB431542 resulted in a higher expression of cementoblast markers compared to treatment with SB431542 alone (Figure 1(a), SB and B7/SB in A–E). The transcription levels of two representative cementoblastic markers, cementum attachment protein (CAP) and cementum protein-1 (CEMP1), were increased by 4.4 and 6.5 times, respectively, compared to PDL fibroblastic cells induced by TGF-*β*1 (Figure 1(a), B7/SB and TGF in A and B). The transcription levels of osterix (OSX), osteocalcin (OCN), and bone sialoprotein (BSP) were also increased by 11.1, 4.3, and 50.0 times, respectively, compared to those of fibroblastic cells (Figure 1(a), BMP7/SB and TGF in C–E). Conversely, when cells were treated with TGF-*β*1, the gene expression of PDL fibroblastic markers scleraxis (SCX) and periodontal ligament-associated protein-1 (PLAP-1) were increased by 7.7 and 5.2 times, respectively, compared to that of cementoblast-like cells (Figure 1(a), BMP7/SB and TGF in F and G). Previous studies showed that OSX played an essential role in osteo/cementoblastic differentiation and that SCX antagonized OSX activity in PDLCs [34, 35]. As shown in Figure 1(a), since OSX and SCX were expressed inversely and representative cementoblast markers were transcriptionally increased, we considered that hPDLCs were differentiated into cementoblast-like cells.

### 3.2. Anti-CM3 Antibody, a Novel Monoclonal Antibody, Recognizes an ~30 kDa Cementoblast-Specific Antigen

We developed a set of monoclonal antibodies against the membrane/ECM molecules of cementoblast-like cells. hPDLCs treated with TGF-*β*1 were used as decoy controls. This study used a novel IgG-type antibody, the anti-CM3 antibody, to identify the cementoblast-specific antigen. Flow cytometry showed that the cell-binding affinity of the anti-CM3 antibody was highly increased in hPDLCs treated with BMP7 and SB431542 (Figure 1(b), B7/SB in A). The anti-CM3 antibody exhibited cell-binding affinity in an immortalized murine cementoblast cell line (OCCM-30) (Figure 1(b), OCCM in B). An endogenous antigenic molecule recognized by the anti-CM3 antibody was increased in both human cementoblast-like cells differentiated from hPDLCs and murine cementoblast OCCM-30 (Figures 1(c), A and B). Immunohistochemical analysis revealed that the antigenic protein recognized by the anti-CM3 antibody was more abundant in the cementum region, a thin layer situated just outside the dentin, than in the broad PDL region in human tooth slices (Figure 1(d)). As expected, anti-OSX and anti-PLAP-1 antibodies accumulated in the cementum and PDL (Figures 1(d), C and D). These results suggest that the antigen recognized by the anti-CM3 antibody may be a cementoblast-specific cellular marker.

**Figure 2:**
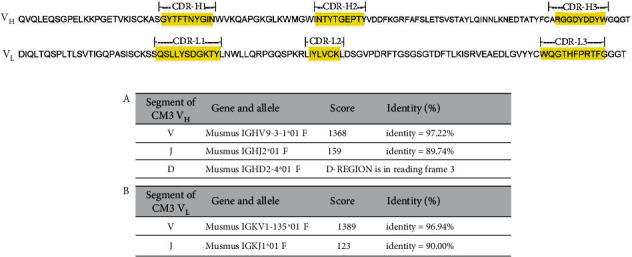
Characterization of anti-CM3 monoclonal antibody. (a) Amino acid sequence of variable regions of heavy and light chains. Three complementary determining regions (CDRs) in heavy and light chains (CDR-L1~3 and CDR-H1~3) are shown in shading boxes. (b) Results of the IMGT/V-QUEST analysis of the anti-CM3 antibody. Identity indicated percentage of nucleotides identical to the reference sequence. (A) IMGT/V-QUEST analysis of *V*_*H*_; (B) IMGT/V-QUEST analysis of *V*_*L*_.

cDNA sequencing of the variable antibody regions and IMGT/V-QUEST database-based antibody similarity analysis defined the complementarity-determining regions (CDRs) of the anti-CM3 antibody [36, 37]. Amino acid sequences around the variable chains of the CDRs were observed to be well-conserved (Figure 2(a)). The V and J segments of the heavy chain were found to share 97.22% and 89.74% similarity with Musmus IGHV9-3-1^∗^01 F and Musmus IGHJ2^∗^01 F, respectively. The V and J segments of the light chain were found to share 96.94% and 90.00% similarity with Musmus IGKV1-135^∗^01 F and Musmus IGKJ1^∗^01 F, respectively (Figure 2(b)). These data indicate that the anti-CM3 antibody is a novel monoclonal antibody belonging to the IgG1 and IG*κ*-V1 heavy and light chain subgroups.

### 3.3. The Antigenic Molecule of Anti-CM3 Antibody Is Galectin-3

**Figure 3:**
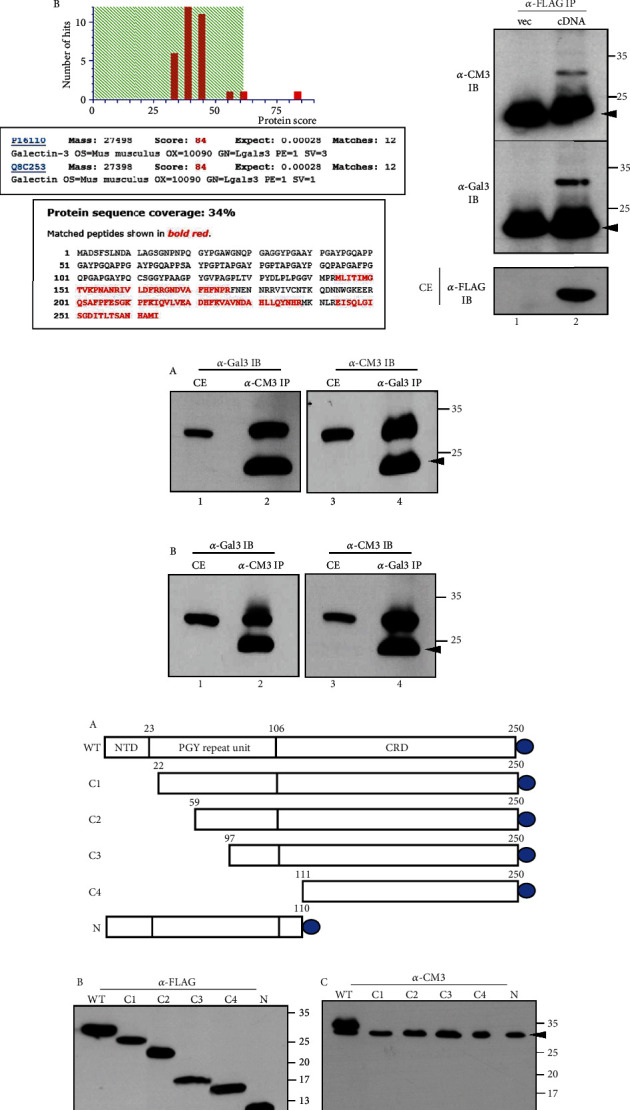
The antigenic molecule recognized by the anti-CM3 antibody turns out to be galectin-3. (a) Identification of the antigenic molecule recognized by the anti-CM3 antibody. (A) Detection of the CM3 antigenic molecule. The antigenic molecule labeled with biotin was detected by HRP-conjugated streptavidin (left panel) and by staining with Coomassie Blue (right panel). Lane 1, protein G beads from preclearing; lanes 2 and 3, immunoprecipitates with anti-CM3 antibody; lane 4, protein size marker. The antigenic molecule was indicated by an arrow. (B) Tandem mass spectrometric data of anti-CM3 immunoprecipitates. (b, c) Galectin-3 protein is recognized by the anti-CM3 antibody. Ectopically expressed galectin-3 containing FLAG tag and endogenous galectin-3 were detected by the anti-CM3 antibody. (b) Anti-FLAG immunoprecipitates (*α*-FLAG IP) were recognized by both anti-CM3 and anti-galectin-3 antibodies. Lane 1, vector only; lane 2, cDNA transfection. (c) Anti-CM3 antibody has cross-reactivity with commercially available anti-galectin-3 antibody. Anti-CM3 immunoprecipitates (*α*-CM3 IP) were recognized by anti-galectin-3 antibody (*α*-Gal3 IP) and vice versa in BMP7-induced human cementoblasts (A) and the mouse cementoblast cell line OCCM-30 (B). Lanes 1 and 3, protein amounts in cell extract; lanes 2 and 4, immunobinding in immunoprecipitates. The arrowheads indicate the light chain of IgG. (d) Epitope mapping of galectin-3 recognized by the anti-CM3 antibody. (A) The schematic presentation of galectin-3 domain constructs. All constructs tagged with FLAG (circle) were expressed in HeLa cells and were detected by anti-FLAG (B) and anti-CM3 (C) antibodies. The arrowhead in (C) indicates endogenous galectin-3 expressing in HeLa cells.

Immunoprecipitation using cell extracts prepared from biotin-labeled OCCM-30 was performed to determine the antigenic protein recognized by the anti-CM3 antibody. A specific band between the 25 and 35 kDa of protein markers was strongly detected in anti-CM3 immunoprecipitates using a streptavidin-horseradish peroxidase-conjugated secondary antibody (Figure 3(a), arrow in lane 2 in A). Excision of the protein band from the gel (Figure 3(a), arrow in lane 3 in A) and analysis via tandem mass spectrometry identified the protein as galectin-3 with 34% protein sequence coverage (Figure 3(a), B).

**Figure 4:**
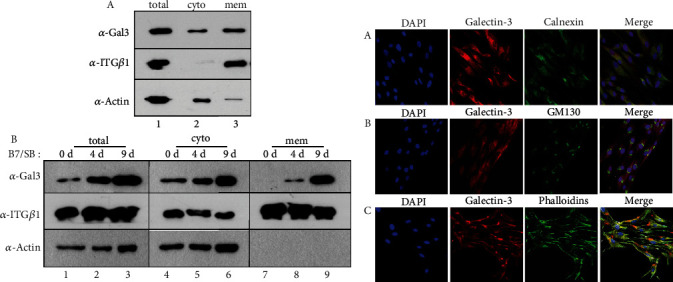
Galectin-3/CM3 was accumulated in cell membrane fraction during BMP7-induced cementoblastic differentiation. For induction of cementoblastic differentiation, hPDLCs were treated with 100 ng/mL BMP7 and 10 *μ*M SB431542 for 9 days. Cells were harvested at the indicated times, and cytosolic and membrane fractions were prepared as mentioned in Materials and Methods. (a) Galectin-3 was detected in both cytosolic and membrane fractions in mouse cementoblasts (A) and in BMP7-induced human cementoblast-like cells (B). total: total extract; cyto: cytosolic fraction; mem: membrane fraction. In (B), BMP7 and SB431542 (B7/SB) were treated in hPDLCs for 0 day (lanes 1, 4, and 7), 4 days (lanes 2, 5, and 8), and 9 days (lanes 3, 6, and 9). Integrin *β*1 (ITG*β*1) was used as a marker for membrane fraction in western blots. (b) Subcellular localization of galectin-3/CM3 in BMP7-induced human cementoblast-like cells. (A) Immunostaining with Cy3-galectin-3 and FITC-calnexin; (B) immunostaining with Cy3-galectin-3 and FITC-GM130; (C) immunostaining with Cy3-galectin-3 and FITC-phalloidin.

The full-length cDNA construct of human galectin-3 containing a C-terminal FLAG tag was cloned into a mammalian expression vector and ectopically expressed in HeLa cells. The ectopically expressed galectin-3 could be detected by an anti-FLAG antibody (Figure 3(b), lane 2 in *α*-FLAG IB). Immunoprecipitates of the anti-FLAG antibody were strongly recognized by both the anti-CM3 antibody and the anti-galectin-3 antibody (purchased from Santa Cruz, raised against amino acids 1-18) (Figure 3(b), lane 2 in *α*-FLAG IB and *α*-Gal3 IB). In addition to the ectopic expression of galectin-3 cDNA, the endogenous galectin-3 protein cross-reacted with anti-CM3 and anti-galectin-3 antibodies in OCCM-30 and human cementoblast-like cells, respectively (Figures 3(c), A and B). Immunoprecipitates containing the anti-CM3 antibody were strongly recognized by the anti-galectin-3 antibody and vice versa (Figure 3(c), lanes 2 and 4 in A and B). To verify the epitope region in galectin-3 recognized by the anti-CM3 antibody, the domain constructs of galectin-3 were expressed in HeLa cells (Figure 3(d), A). A short N-terminal domain (NTD) containing a serine phosphorylation site is known to be involved in the oligomerization of galectin-3. The carbohydrate recognition domain (CRD), consisting of 130 amino acids, comprises the C-terminal and contains an Asn-Trp-Gly-Arg (NWGR) motif [38, 39]. In addition to the full-length protein (WT), all the domain constructs were detected by the anti-FLAG antibody (Figure 3(d), B). However, the anti-CM3 antibody only recognized the full-length and N-terminal domain constructs, not the C-terminal deletion constructs (Figure 3(d), C). Endogenous galectin-3 proteins present in the HeLa cells were also detected by the anti-CM3 antibody (Figure 3(d), arrowhead in lanes 1–6 in C). These results indicated that the CM3 antigenic protein was identical to galectin-3. In mouse cementoblast OCCM-30, galectin-3/CM3 was detected in both the cytosolic and membrane fractions (Figure 4(a), A). During cementoblastic differentiation of hPDLCs, the amount of galectin-3/CM3 gradually increased in total cell extracts (Figure 4(a), lanes 1–3 in B). Although galectin-3/CM3 also increased gradually in both the cytosolic and membrane fractions during differentiation, it was present in the cytoplasm regardless of differentiation status (Figure 4(a), lanes 4–6 in B). However, it was completely absent from the membrane in undifferentiated cells and was found to be highly accumulated in the membrane fraction during cementoblastic differentiation (Figure 4(a), lanes 7–9 in B). Immunocytochemical analysis showed that galectin-3/CM3 was widespread throughout the cells, independent of the endoplasmic reticulum (ER) and Golgi regions (Figure 4(b), A and B). Without membrane permeabilization, galectin-3/CM3 was located at the edge of the cell via phalloidin staining, which can be inferred as the cell boundary (Figure 4(b), C). This indicated that galectin-3/CM3 is present in the cytoplasm and membrane regions of the cell margin in cementoblast-like cells.

### 3.4. Ectopic Expression of Galectin-3 Increases the Efficiency of Cementoblastic Differentiation

**Figure 5:**
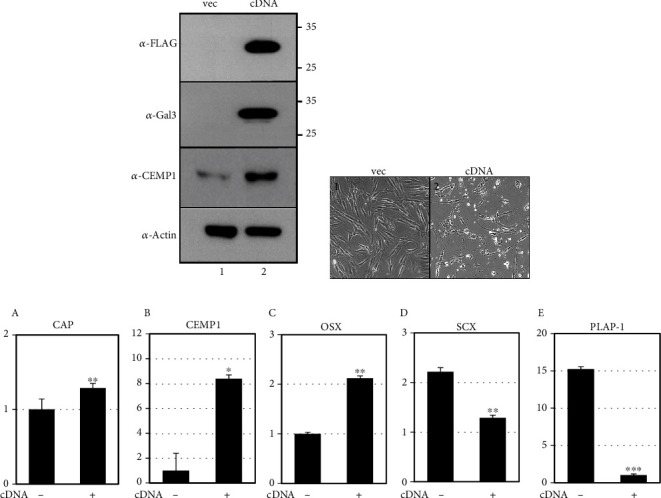
Ectopic expression of galectin-3 inhibits fibroblastic activation and induces cementoblastic differentiation. Ectopic expression of full-length cDNA of galectin-3 in hPDLCs was performed as indicated in Materials and Methods. The changes of protein amounts and cellular morphologies are shown in (a, b), respectively. 1, vector only; 2, cDNA transfection. (c) Transcriptional expression of the representative cementoblastic (a–c) and fibroblastic (d, e) markers. ^∗^*P* < 0.05; ^∗∗^*P* < 0.01; ^∗∗∗^*P* < 0.001. Statistical analysis was performed using Student's *t*-test (*n* = 3).

To investigate the role of galectin-3/CM3 in cementoblastic differentiation, the full-length protein of galectin-3 was overexpressed ectopically in hPDLCs. Because the cDNA of galectin-3 was tagged with a FLAG epitope, the ectopic protein was detected by the anti-FLAG antibody (Figure 5(a), *α*-FLAG). Although some cells tend to die when overexpressed, cementoblastic differentiation was shown to be induced (Figure 5(b)). The CEMP1 protein level (Figure 5(a), *α*-CEMP1) and the transcriptional expression of the representative cementoblastic markers were both increased (Figure 5(c), A–C). Interestingly, the overexpression of galectin-3 lowered the expression of the SCX and PLAP-1 fibroblastic genes in hPDLCs (Figure 5(c), D and E).

### 3.5. Inhibition of Galectin-3 via siRNA and the Blocking of Cementoblast Differentiation Using the Specific Inhibitor GB1107

**Figure 6:**
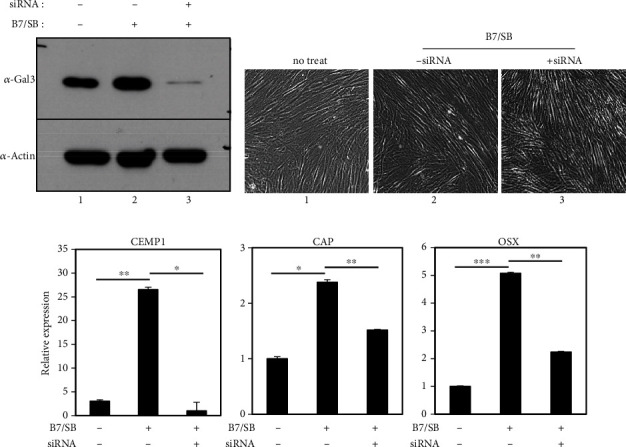
Depletion of galectin-3/CM3 in hPDLSCs inhibits BMP7-induced cementoblastic differentiation. Endogenous galectin-3 was depleted using siRNA construct as indicated in Materials and Methods. The protein levels of endogenous galectin-3 and the cell morphologies are shown in (a, b). 1, undifferentiated hPDLCs; 2, hPDLCs treated with BMP7 and SB431542 for 9 days; 3, hPDLC treated simultaneously with BMP7, SB431542, and siRNA constructs. (c) Transcriptional expression of the representative cementoblastic markers. Statistical significance is determined using Student's *t*-test (*n* = 3). ^∗^*P* < 0.05; ^∗∗^*P* < 0.01; ^∗∗∗^*P* < 0.001.

**Figure 7:**
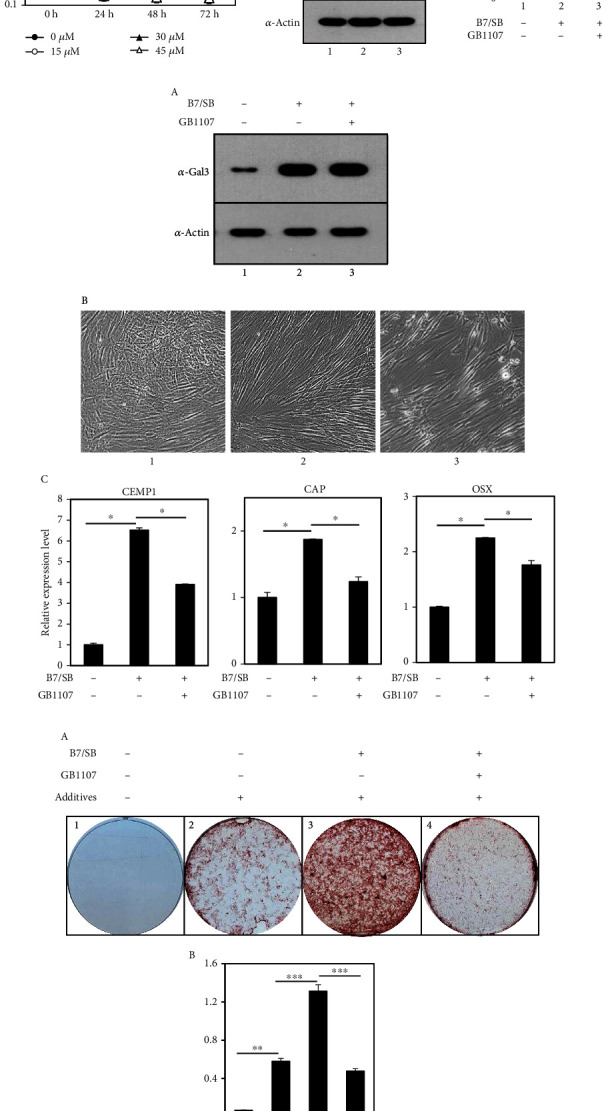
Treatment with GB1107, a galectin-3-specific inhibitor, inhibits BMP7-induced cementoblastic differentiation. (a) hPDLCs were treated with the indicated concentrations of GB1107 for up to 72 hours. Cells were collected at the indicated time, and the proliferation assay was performed to assess the cells' viability, as indicated in Materials and Methods. (b) Downregulation of BMP7-Smad1 signaling via treatment with GB1107. The amounts of the indicated proteins and the relative values of p-Smad1 to Smad1 are shown in (A) and (B). In (A), lane 1, undifferentiated hPDLCs; lane 2, hPDLCs treated with 100 ng/mL BMP7 and 10 *μ*M SB431542 for 72 hours; lane 3, hPDLCs treated with BMP7, SB431542, and 15 *μ*M GB1107 for 72 hours. In (B), the quantification of the intensity of each band on immunoblots and the calculation of relative values of p-Smad1 (indicated by arrowhead) to Smad1 were analyzed using ImageJ program [60]. (c) GB1107 inhibited BMP7-induced cementoblastic differentiation. When 15 *μ*M GB1107 was continuously treated during the 9-day differentiation period, the amount of endogenous galectin-3 (A) and cell morphologies (B) were not affected. In (A), lane 1, undifferentiated hPDLCs; lane 2, hPDLCs treated with 100 ng/mL BMP7 and 10 *μ*M SB431542 for 9 days; lane 3, hPDLCs treated with BMP7, SB431542, and 15 *μ*M GB1107 for 9 days. Transcriptional expression of the representative cementoblastic markers is shown in (C). ^∗^*P* < 0.05. Statistical analysis was performed using Student's *t*-test (*n* = 3). (d) Inhibition of galectin-3 downregulated mineralization efficiency of human cementoblast-like cells. After 14-day incubation in media containing mineralization additives, degree of mineralization was detected using alizarin red S staining (A) and was quantified via optical density at 405 nm (B). ^∗∗^*P* < 0.01; ^∗∗∗^*P* < 0.001. Statistical analysis was performed using Students *t*-test (*n* = 3).

When endogenous galectin-3 was depleted using siRNA constructs, the effect of BMP7 on the induction of cementoblast differentiation in hPDLCs was completely reduced (Figure 6). Although there was little effect on the shape and proliferation of the cells following galectin-3 depletion (panels 2 and 3 in Figures 6(a) and 6(b)), the expression of the representative markers CEMP1, CAP, and OSX decreased in a manner similar to that of the control group (Figure 6(c)). GB1107 is a monosaccharidic galectin-3 inhibitor that targets the galectin CRD in a complex with lactose [40]. hPDLCs were treated with various concentrations of GB1107 at different times to determine the optimal conditions. Cell proliferation was used to determine the optimal inhibiting condition of 10~15 *μ*M (Figure 7(a)). Following the inhibition of galectin-3 via GB1107 treatment for 2 days, the phosphorylation of Smad1 by BMP7 was reduced to control levels (Figure 7(b), lane 3 in A and B). GB1107 treatment did not change the amount of endogenous galectin-3, and although a slower growth rate was observed, cell survival was not significantly affected (Figure 7(c), lanes 2 and 3 in A and panel 3 in B). However, the transcription levels of the cementoblastic markers were decreased via GB1107 treatment (Figure 7(c), C). Additionally, mineralization induction using BMP7 was greatly reduced after galectin-3 inhibition (Figure 7(d), panels 3 and 4 in A). These results suggest that galectin-3 expression played an important role in BMP7-induced cementoblast differentiation.

### 3.6. GB1107, Targeting the CRD of Galectin-3, Is Inhibited by the Interaction of Galectin-3 with Laminin *α*2 and BMP7

**Figure 8:**
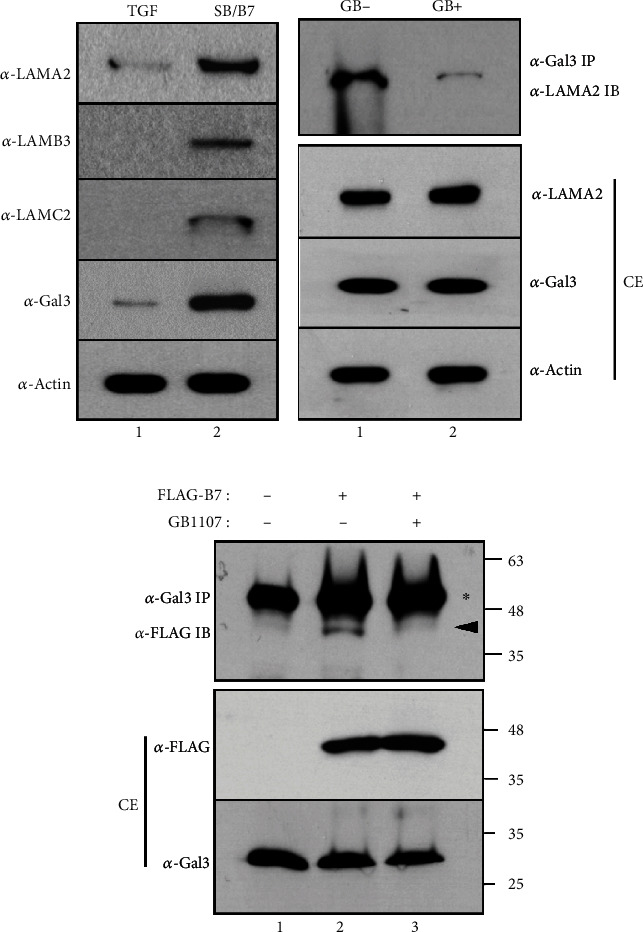
Galectin-3 interacts with laminin *α*2 or BMP7 in cementoblast-like cells. (a) Endogenous protein level of laminins in TGF-*β*1-induced PDL fibroblasts (lane 1) and BMP7-induced cementoblast-like cells (lane 2). LAMA2: laminin *α*2; LAMB3: laminin *β*3; LAMC3: laminin *γ*3. (b) GB1107 effect on the interaction between galectin-3 and laminin *α*2. Laminin *α*2 was detected in the immunoprecipitates of galectin-3 (*α*-Gal3 IP/*α*-LAMA2 IB). Endogenous protein amounts of galectin-3 and laminin *α*2 in total cell extracts (CE) were not changed due to GB1107 treatment. Lane 1, no treatment with GB1107 (-GB); lane 2, treatment with GB1107 (+GB). (c) GB1107 effect on the interaction between galectin-3 and ectopic BMP7. BMP7 expressed ectopically was detected in the immunoprecipitates of galectin-3 (*α*-Gal3 IP/*α*-FLAG IB). Endogenous protein amounts of galectin-3 and ectopic BMP7 in HeLa cell extracts (CE) were not changed due to GB1107 treatment. Lane 1, no expression of BMP7; lane 2, cells with BMP7 cDNA expression without GB1107 treatment; lane 3, cells with BMP7 cDNA expression with GB1107 treatment.

Laminins are essential structural noncollagenous glycoproteins that are localized to cell-associated ECMs and guide cell differentiation, proliferation, and tissue architecture. Each laminin is a heterotrimer of *α*, *β*, and *γ* chain subunits and undergoes cell secretion and incorporation into the ECM [41]. We recently reported that the transcriptional expression of laminins was markedly increased in cementoblast-like cells differentiated from hPDLCs via induction with BMP7, which is contrary to the increased expression of collagens in ligament fibroblasts [16]. Since galectin-3 interacts with the *β*-galactoside residues of laminins through the CRD to exert effects on the modulation of cell adhesion [42], we investigated the interaction between laminins and galectin-3. When hPDLCs were differentiated into cementoblast-like cells, the expression of endogenous laminin *α*2, laminin *β*3, and laminin *γ*3 was highly increased (Figure 8(a), lane 2). In contrast, their expression was barely detectable in PDL fibroblasts (Figure 8(a), lane 1). Coimmunoprecipitation revealed that galectin-3 was associated with laminin *α*2 (Figure 8(b), lane 1 in *α*-Gal3 IP/*α*-LAMA2 IB). The association was completely inhibited by the presence of GB1107, although the expression level of laminin *α*2 was similar in cells untreated with an inhibitor (Figure 8(b), lane 2 in *α*-Gal3 IP/*α*-LAMA2 IB). Since galectins bind glycosylated cytokines and retain them within the ECM [38, 39], we examined whether galectin-3 would bind to BMP7. Full-length cDNA of BMP7, tagged with a FLAG peptide, was ectopically expressed in HeLa cells, and the interaction between endogenous galectin-3 and ectopic BMP7 was examined via coimmunoprecipitation. The anti-FLAG antibody recognized BMP7 in the immunoprecipitates of galectin-3 (Figure 8(c), lane 2 in *α*-Gal3 IP/*α*-FLAG IB), and the interaction between galectin-3 and BMP7 was not evident after GB1107 treatment (Figure 8(c), lane 3 in *α*-Gal3 IP/*α*-FLAG IB).

## 4. Discussion

The periodontal ligament (PDL), located between the alveolar bone and the cementum of the tooth, is part of the connective tissue that secures the tooth root to the bone. Multipotent PDL stem cells, capable of differentiating into multiple progenitor cells, were found in PDL tissue [43, 44]. These postnatal stem cells give rise to both the cementum and PDL fibers and regenerate periodontium by restoring periodontal defects [45, 46], suggesting that PDL stem cells may differentiate into both cementoblasts and PDL fibroblasts. Therefore, the differentiation of PDL stem cells into mineralized cementum and nonmineralized ligament should be well-coordinated. Previous studies reported that BMP7 treatment accompanied by the inhibition of TGF-*β*1 signaling had a synergistic effect on cementoblastic differentiation. BMP7 induces the expression of the cementogenic markers in hPDLSCs in a completely different way from odontogenic differentiation [4, 12, 13]. To discover cell surface factors that are specifically expressed during cementoblastic differentiation, we initially investigated novel membrane/cell surface molecules of cementoblast-like progenitors through decoy immunization. We identified galectin-3 as a specific antigen recognized by a novel anti-CM3 antibody. Galectin-3 was shown to be indispensable for normal bone cell differentiation and bone remodeling in mice [47]. In recent reports, the expression of galectin-3 improved after osteogenic differentiation, although galectin-3-mediated osteogenic differentiation was partly dependent on favorable partner proteins, such as LGALS3BP and TRIM16 in hPDLSCs and hBMSCs [24, 48]. In addition to these previous reports, we showed that the upregulation of galectin-3 enhanced cementoblastic differentiation after BMP7 treatment of hPDLSCs and suppressed PDL fibroblastic differentiation (Figure 5).

The epitope region recognized by the anti-CM3 antibody was located at the N-terminal nonlectin domain (Figure 3(d)), suggesting that this novel antibody is suitable for coimmunoprecipitation studies on the extracellular ligands of galectin-3 since the carbohydrate recognition and binding functions of galectin-3 were not affected by antibody binding. Although high concentrations of the anti-CM3 antibody were added to the cementoblast induction medium, the differentiation efficiency was not affected (data not shown). This is contrary to the results where the galectin-3 inhibitor, GB1107, and galectin-3 siRNA were used to inhibit cementoblastic differentiation (Figures 6 and 7) and suggests that the N-terminal region does not play an essential role in the effect of galectin-3 on differentiation.

**Figure 9:**
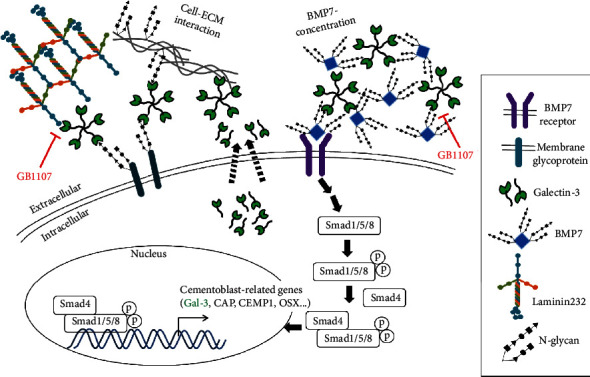
Schematics of galectin-3 function during BMP7-mediated cementoblastic differentiation. Cytosolic galectin-3 is secreted by a nonclassical mechanism via exosomes, because galectins lack a signal peptide (dotted line) [61]. Once in the extracellular space, galectin-3 can interact with innumerous glycosylated molecules, such as cell adhesion molecules, integrins, and ECM molecules such as laminins. Additional galectin-3 partner is glycosylated BMP7, and galectin-3 may gather BMP7 in extracellular space. According to long-term stimulation by BMP7, galectin-3 expression is highly increased, and the differentiation efficiency is amplified due to cell-ECM interaction and BMP7 concentration. GB1107, a galectin-3 inhibitor, reduces the efficiency of cementoblastic differentiation by inhibiting the interaction of laminin and BMP7 with galectins.

The unique extracellular environment, originating from variations in the composition and function of ECM components, is known to regulate stem cell fate [49]. ECM proteins have been shown to guide the differentiation of stem cells [50, 51]. For example, the incubation of PDL stem cells with the ECM membrane isolated from porcine urinary bladders increased the expression of cemento/osteogenic differentiation markers [52]. Extracellular galectin-3 participates in binding to the ECM component laminin to regulate cell adhesion during cell growth and differentiation [53–56]. Since the unique laminin complex LN-332, containing laminin *α*3, *β*3, and *γ*2 chains, can bind to BMP2 through cysteine-rich regions in osteogenic differentiation, it has a crucial effect on the role of ECM as storage for BMPs during differentiation [57–59]. Our recent RNA-seq study reported that galectin-3 and laminin *α*2 expression was increased in cementoblast-like cells following BMP7 treatment [16]. Unexpectedly, the expression of laminin *α*3, one of the three chains of the osteoblastic laminin, was not detected in cementoblast-like cells (data not shown). As shown in Figure 8, galectin-3 interacted with laminin *α*2, and this interaction was completely disrupted by a galectin-3 inhibitor. Unlike laminin *α*2, laminin *β*3 and *γ*2 chains did not interact with galectin-3 (data not shown), although their expressions were highly increased during cementoblastic differentiation (Figure 8(a)). These results suggest that this type of laminin complex may act differently in BMP2-induced osteoblastic and cementoblastic differentiation. That is, it showed that the osteoblastic laminin complex could be LN332, and the cementoblastic laminin complex could be LN232. In addition to the laminin complex, galectin-3 also participates in trapping corresponding cytokines or growth factors to be released in a sustained fashion for the regulation of cell growth and differentiation [38, 39, 41, 58]. In fact, BMP7, a glycoprotein with three N-glycosylation sites, interacted with galectin-3 (Figure 8(c)). The actions of galectin-3 during cementogenic differentiation by BMP7 are summarized in Figure 9.

In conclusion, a novel antibody recognizing intact cementoblast-like cells as an antigen revealed galectin-3 as an important molecule for cementoblastic differentiation. Although little information regarding the factors involved in the mechanism by which PDL stem cells are differentiated into cementoblasts currently exists, the results of this study revealed important facts. Because cell surface and extracellular molecules, which increase during cementoblastic differentiation, were elucidated in the antibody screening strategy, this study focused on the function of extracellular galectin-3. The study findings showed that galectin-3 plays an important role in the differentiation of hPDLCs into cementoblasts through interaction with a specific type of laminin or cytokine BMP7 on the cell surface.

## Figures and Tables

**Table 1:** Primers used for the quantitative real-time PCR (qPCR).

Gene		Primer sequence
GAPDH	Forward Reverse	5′-GTATGACAACAGCCTCAAGAT-3′ 5′-CCTTCCACGATACCAAAGTT-3′

Cementum attachment protein (CAP)	Forward Reverse	5′-TCCAGACATTTGCCTTGCTT-3′ 5′-TTACAGCAATAGAAAAACAGCAT-3′

Cementum protein-1 (CEMP1)	Forward Reverse	5′-GATCAGCATCCTGCTCATGTT-3′ 5′-AGCCAAATGACCCTTCCATTC-3′

Osterix (OSX)	Forward Reverse	5′-GAAGGGAGTGGTGGAGCCAAAC-3′ 5′-ATTAGGGCAGTCGCAGGAGGAG-3′

Osteocalcin (OCN)	Forward Reverse	5′-TGAGTCCTGAGCAGCAG-3′ 5′-TCTCTTCACTACCTCGCT-3′

Bone sialoprotein (BSP)	Forward Reverse	5′-TACCGAGCCTATGAAGATGA-3′ 5′-CTTCCTGAGTTGAACTTCGA-3′

Scleraxis (SCX)	Forward Reverse	5′-AGAAAGTTGAGCAAGGACC-3′ 5′-CTGTCTGTACGTCCGTCT-3′

Periodontal ligament-associated protein-1 (PLAP-1)	Forward Reverse	5′-TTGACCTCAGTCCCAACCAA-3′ 5′-TCGTTAGCTTGTTGTTGTTCAG-3′

## Data Availability

The data that support the findings of this study are available from the corresponding author upon reasonable request.
